# Alterações Ecocardiográficas da Geometria e da Função Cardíaca em Pacientes com Lipodistrofia Familiar Parcial

**DOI:** 10.36660/abc.20230442

**Published:** 2024-06-06

**Authors:** Minna Moreira Dias Romano, André Timóteo Sapalo, Natália Rossin Guidorizzi, Henrique Turin Moreira, Paula Ananda Chacon Inês, Lucas Candelária Kalil, Maria Cristina Foss, Francisco José Albuquerque de Paula

**Affiliations:** 1 Universidade de São Paulo Centro de Cardiologia da Faculdade de Medicina de Ribeirão Preto São Paulo Brasil Centro de Cardiologia da Faculdade de Medicina de Ribeirão Preto – Universidade de São Paulo (USP), São Paulo – Brasil; 2 Universidade de São Paulo Divisão de Endocrinologia da Faculdade de Medicina de Ribeirão Preto São Paulo Brasil Divisão de Endocrinologia da Faculdade de Medicina de Ribeirão Preto – Universidade de São Paulo (USP), São Paulo – Brasil

**Keywords:** Lipodistrofia, Testes de Função Cardíaca, Ecocardiografia

## Abstract

**Fundamento:**

A cardiomiopatia associada à lipodistrofia parcial (LP) ainda não foi bem descrita.

**Objetivo:**

Caracterizar a morfologia e a função cardíaca na LP.

**Métodos:**

Pacientes com LP e controles foram avaliados prospectivamente por ecocardiografia transtorácica e ecocardiografia por *speckle-tracking* (*Strain* Longitudinal Global, SLG). A relação entre as variáveis ecocardiográficas e o diagnóstico de LP foi testada com modelos de regressão, considerando o efeito da pressão arterial sistólica (PAS). Adotou-se um nível de significância de 5%.

**Resultados:**

Vinte e nove pacientes com LP foram comparados com 17 controles. Eles não se diferiram quanto à idade (p=0,94), sexo ou índice de massa corporal (p= 0,05). Os pacientes com LP apresentaram PAS estatisticamente mais alta (p=0,02) em comparação aos controles. Ainda, os pacientes com LP apresentaram maior dimensão do átrio (37,3 ± 4,4 vs. 32,1 ± 4,3 mm, p= 0,001) e maior volume atrial (30,2 ± 7,2 vs. 24,9 ± 9,0 mL/m^2^, p=0,02), massa do Ventrículo Esquerdo (VE) (79,3 ± 17,4 vs. 67,1 ± 19,4; p=0,02), e parâmetros sistólicos reduzidos do VE (E’ lateral, p= 0,001) (E’ septal, p= 0,001), (razão E/E’, p= 0,02). A fração de ejeção do VE (64,7 ± 4,6 vs. 62,2 ± 4,4 %, p = 0,08) e o SLG não foram estatisticamente diferentes entre os grupos (-17,1±2,7 vs-18.0 ± 2,0%, p= 0,25). Observou-se uma reação positiva do átrio esquerdo (*β* 5,6; p<0,001), espessura da parede posterior (*β* 1,3; p=0,011), E’ lateral (*β* -3,5; p=0,002) e E’ septal (*β* -3,2; p<0,001) com o diagnóstico de LP, mesmo após o ajuste para a PAS.

**Conclusão:**

Os pacientes com LP apresentam hipertrofia do VE, aumento do átrio esquerdo, e disfunção diastólica do VE apesar de fração de ejeção do VE e SLG preservados. Os parâmetros ecocardiográficos estão relacionados com o diagnóstico de LP, independentemente da PAS.

**Figure:**
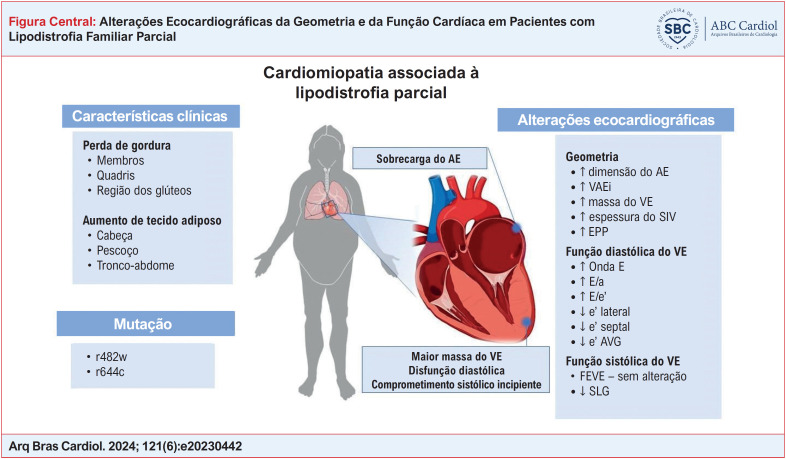


## Introdução

A lipodistrofia parcial (LP) é uma condição rara caracterizada pela perda de tecido adiposo de forma generalizada ou parcial.^1^ Estima-se que a prevalência de lipodistrofia seja de 1,3 a 4,7 casos por milhão de pessoas, sendo mais alta em populações consanguíneas.^2^ Sua etiologia pode ser congênita^3^ ou adquirida, e ambas envolvem uma deficiência na produção do hormônio leptina.^4^ A deposição ectópica de tecido adiposo e de triglicerídeos causa complicações como resistência à insulina, diabetes mellitus, hipertrigliceridemia, esteatose hepática, e risco aumentado de doença cardiovascular. A LP familiar pode resultar de variantes patogênicas do gene LMNA. Embora as consequências da deficiência de leptina ainda sejam mal compreendidas,^5,6^ o tratamento com leptina exógena pode ser altamente eficaz^7,8^ em alguns casos de lipodistrofia generalizada.

Apesar de a doença cardiovascular ser uma causa importante de morte precoce nessa população,^9^ a cardiomiopatia associada à LP ainda não foi bem descrita. A aterosclerose precoce, principalmente em pacientes com LP familiar, pode ter uma prevalência superior a 60% e se manifestar antes dos 45 anos de idade.^10^ Os mecanismos fisiopatológicos parecem depender não só das alterações metabólicas como também de um efeito direto da mutação genética sobre a função endotelial.^4,11^ Alguns casos de lipodistrofia foram relatados com hipertrofia do ventrículo esquerdo (VE), outros com características de dilatação do VE. Muitos foram associados com estados pró-inflamatórios,^12-16^ similar a outras condições metabólicas relacionadas à insuficiência cardíaca com fração de ejeção do VE preservada.^17^ Pacientes com LP por mutações no gene LMNA apresentam um risco aumentado para arritmias.

A ecocardiografia é uma ferramenta não invasiva capaz de caracterizar alterações morfológicas e funcionais cardíacas. Medidas convencionais das dimensões da câmara cardíaca, da função ventricular sistólica e diastólica, e técnicas novas de avaliação da deformação miocárdica, tais como ecocardiografia com *speckle tracking*,^18,19^ foram capazes de detectar alterações precoces no miocárdio em pacientes com LP generalizada,^20^ mas ainda não foram estudadas em pacientes com LP familiar. Assim, neste estudo, nosso objetivo foi caracterizar a morfologia cardíaca e disfunção inicial do VE em um grupo de pacientes com LP familiar sem sintomas cardíacos.

## Métodos

### População do estudo

Este é um estudo transversal que comparou casos e controles. Uma amostra (de conveniência) de pacientes com diagnóstico clínico de LP familiar foi convidada para realizar uma análise ecocardiográfica prospectiva. O diagnóstico clínico de LP familiar baseou-se em uma apresentação fenotípica de distribuição de gordura corporal, associada a alterações metabólicas como disglicemia e hipertrigliceridemia. Alguns pacientes, com base na indicação clínica, haviam se submetido a teste genético pelo método de Sanger ou painéis genéticos (painel de sequenciamento de nova geração, NGS, do inglês *Next Generation Sequencing*). Voluntários não afetados, pareados (1:1) por sexo e idade foram convidados para participar como grupo controle. Os pacientes foram recrutados de uma clínica de endocrinologia, onde foram realizados a avaliação clínica e os testes bioquímicos e genéticos. Os pacientes com diagnóstico de LP e com idade maior que 18 anos foram convidados a participar. Os critérios de exclusão foram pacientes com imagens ecocardiográficas de baixa qualidade. Os dados clínicos incluíram idade, sexo, índice de massa corporal (IMC), área de superfície corporal (ASC), história de hipertensão, pressão arterial sistólica (PAS), e pressão arterial diastólica (PAD). Os dados laboratoriais incluíram colesterol total, colesterol LDL, colesterol HDL, triglicerídeos, glicemia de jejum, e HbA1c.

O comitê de ética local aprovou o estudo (número de protocolo HCRP_3.744.254), e todos os participantes assinaram um termo de consentimento antes dos procedimentos do estudo.

### Ecocardiografia

Todas as imagens de ecocardiografia transtorácica foram coletadas prospectivamente para garantir a melhor qualidade da imagem. As imagens foram adquiridas com o sistema Vivid E9 ou E95 (GE Healthcare, Horten, Noruega), com um transdutor de 1,4-4,6 MHz. A aquisição das imagens foi realizada seguindo-se diretrizes publicadas anteriormente.^21^ Em resumo, imagens apicais do VE foram adquiridas no seu eixo mais longo possível, evitando-se, assim, o encurtamento do VE. As imagens foram registradas com os traçados eletrocardiográficos em pelo menos três ciclos cardíacos consecutivos em repouso. Todas as imagens foram adquiridas a uma taxa de 55-90 quadros por segundo e analisadas de modo *offline* com o programa EchoPac (GE Vingmed Ultrasound AS) versão 112. Os seguintes parâmetros da ecocardiografia convencional das dimensões e função do VE foram coletados: dimensão e volume do átrio esquerdo (AE), massa do VE, dimensão linear do VE na diástole e na sístole – diâmetro diastólico final do VE (DDFVE), diâmetro sistólico final do VE (DSFVE) – volume diastólico final do VE (VDFVE), Volume Sistólico Final do VE (VSFVE), e fração de ejeção do VE (VEFE) derivados do método de Simpson modificado, ondas E e A nas imagens de Doppler do fluxo mitral e tempo de desaceleração (TD), e velocidade diastólica anular mitral septal e lateral por ecocardiografia com Doppler tecidual (e’ lateral e e’ septal).

### Análise do *strain* por ecocardiografia bidimensional

Um único médico experiente foi responsável por realizar as análises de *strain* bidimensional usando as três imagens apicais do VE. Sempre que possível, o segundo dos três ciclos cardíacos adquiridos foi escolhido para análise. Todas as medidas de *strain* foram coletadas como mesocárdio, e valores do volume final sistólico foram medidos, evitando-se as medidas de *strain* pós-sistólico. O tempo de referência foi definido manualmente, no início do QRS. Sístole final foi definida como o momento do fechamento aórtico, definido de sinais de Doppler na via de saída do VE quando se faz a medida do *Strain* Longitudinal Global (SLG). A borda endocárdica foi traçada no final da sístole, e a região de interesse ajustada para excluir o pericárdio. Segmentos com traçados persistentemente inadequados foram excluídos da análise. O VE foi dividido em 18 segmentos, e a exclusão de no máximo dois segmentos foi considerada tolerável. Um ponto de corte de -16% de SLG foi considerado para separar subgrupos de pacientes com lipodistrofia, com base em valores normais previamente publicados para esse software.^19^ Todos os valores de *strain* foram expressos em % de mudança.

### Análise estatística

Os dados foram expressos em média ± desvio padrão e aqueles com uma distribuição não normal em mediana e Intervalo Interquartil (IIQ). A distribuição dos dados contínuos foi testada quanto à normalidade usando o teste de Shapiro-Wilk, e variâncias iguais foram avaliadas usando o teste de Bartlett. As variáveis categóricas foram expressas em porcentagens e frequências. Comparações das variáveis contínuas entre os grupos foram realizadas usando o teste t de Student não pareado ou o teste de Mann-Whitney para dados paramétricos e não paramétricos, respectivamente, e o teste do qui-quadrado para dados categóricos. Considerando que alguns pacientes com LP apresentavam valores mais altos de PAS que os controles em nosso estudo, a relação das variáveis ecocardiográficas com o diagnóstico de LP foi ajustada pela PAS usando a análise univariada, e depois nos modelos de regressão linear. Todas as premissas da análise linear multivariada foram preenchidas. O nível de significância foi estabelecido em p<0,05. Todas as análises estatísticas foram realizadas usando o programa GraphPad Prism para Windows v9.4.1 ou o Stata 14.0 (StataCorp, College Station, TX).

## Resultados

Nós incluímos 31 pacientes com LP. No entanto, dois pacientes foram excluídos devido à baixa qualidade das imagens ecocardiográficas. Um teste genético foi realizado em 78,6% dos pacientes com LPD, e todos eles apresentavam variantes no gene LMNA (59,0% R482W, 22,7% R644C, e 18,2%, com uma significância incerta). O grupo controle consistiu de 17 voluntários não afetados, pareados por idade. As características basais dos pacientes e do grupo controle estão descritas na Tabela 1. Os pacientes com LP e o grupo controle não se diferiram quanto à idade, sexo, IMC ou ASC. Contudo, como o esperado, os pacientes com LP apresentaram valores sanguíneos mais altos de lipídios e de glicemia. Pacientes com LP apresentaram PAS estatisticamente mais alta, mas os níveis não atingiram os critérios para hipertensão.

**Tabela 1:** Características clínicas da população do estudo

Variável		Controle	LP	p
n		17	29	
Idade (anos)		43,84 ± 13,32	44,58 ± 11,77	0,94
Sexo masculino (%)		0,00	27,00	
Peso (Kg)		64,33 ± 13,69	68,66 ± 17,92	0,41
IMC (Kg/m^2^)		24,52 ± 5,04	26,73 ± 4,30	0,05
ASC (m^2^)		1,676 ± 0,16	1,70 ± 0,23	0,9
**Variante patogênica**				
	r482w	0	12	
	r644c	0	5	
**Característica clínica**				
	Glicemia (mg/dl)	88,39 (83,50-96,00)	128,50 (89,06-206)	<0,0001*
	HBA1C (%)	5,45 (4,92-5,87)	8,70 (5,8-11,40)	<0,0001*
	HDL (mg/dl)	55,00 (40,50-61,25)	35,61 (31,04-42,32)	0,01*
	LDL (mg/dl)	104 (84-142)	110,00 (70,50-163,65)	0,58
	TG (mg/dl)	86 (67,50-124)	221,80 (162,00-406,40)	<0,0001*
	PAS (mmHg)	111,00 (101-117)	123,5 (112-134)	0,02*
	PAD (mmHg)	69 (62-72)	75(66-82)	0,1
**Medicamentos (%)**				
	Insulina	0	51,7	
	Metformina	0	41,37	
	Gliclazida	0	3,44	
	Estatina	0	41,37	
	Fibratos	0	68,96	
	Anti-hipertensivo	5	68,96	

IMC: índice de massa corporal; ASC: área de superfície corporal; PAS: pressão arterial sistólica; PAD: pressão arterial diastólica; LP: lipodistrofia parcial.

**Figura 1:**
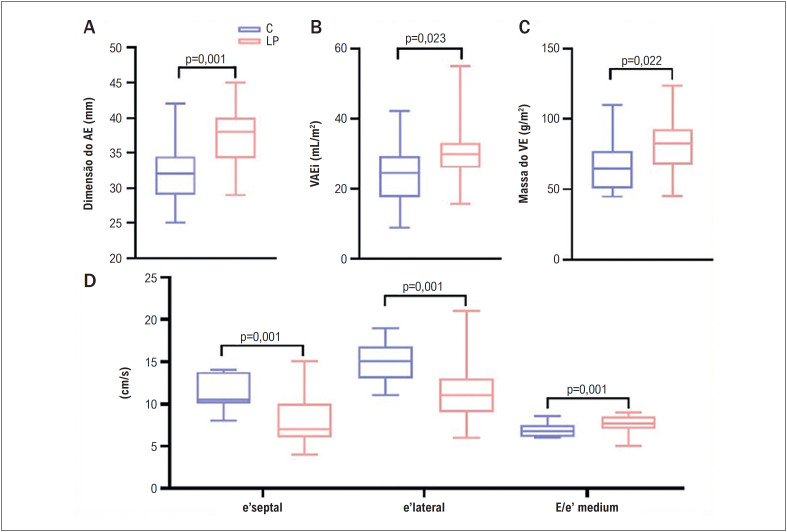
Comparação dos parâmetros ecocardiográficos da geometria cardíaca e função diastólica do ventrículo esquerdo (VE) entre os pacientes com lipodistrofia parcial (LP) e Controles (C); AE: átrio esquerdo; VAEi: volume atrial esquerdo indexado.

Parâmetros ecocardiográficos convencionais da geometria cardíaca e da função do VE estão apresentados na Tabela 2. Algumas variáveis estão expressas como um gráfico nas Figuras 1 e 2.

**Tabela 2:** Características ecocardiográficas da população do estudo

Variável	Controle	LP	p
Dimensão do AE (mm)	32 (28,5-33,75)	38 (34,25-40,00)	0,001*
VAEi (ml/m^2^)	26,26 (20,41-30,89)	30 (26-32,90)	0,023*
massa do VE i(ml/m^2^)	67,54 (49,60-79,79)	77,34 (67,21-88,29)	0,022*
FEVE Simpson (%)	63,50 (59,25-65,75)	64,50 (61-67,50)	0,77
SIV (mm)	8,00 (7-9)	9 (8-11)	0,003*
DDFVE (mm)	44,50 (40,50-48,50)	43 (41-47)	0,526
EPP (mm)	7,5 (6,50-8,87)	9 (8-10)	0,001*
DSFVE (mm)	21,25 (18,13- 24)	20 (15-25)	0,985
VDFVE (ml)	57 (47,93-63)	56 (45,50-77,50)	0,345
E (cm/s)	82 (69-96)	74 (54-83)	0,016*
A (cm/s)	59 (48-73)	69 (53-80)	0,197
E/a	0,891 (0,61-1,54)	2,19 (1,68-3,54)	0,035*
e' lat (cm/s)	15 (12-17)	11 (9-14)	<0,0001
e' sep (cm/s)	10 (10-14)	8 (6-11)	<0,0001
Tempo de desaceleração (mseg)	186 (175,3-217,3)	180 (162-244)	0,45
e´AVG	12,5 (11,5-15,5)	9 (7-12)	<0,0001
E/e´	6,19 (5,15-7,55)	7,38 (6-9,1)	0,017*
SLG (%)	-18,05 (-19,78- - 17,09)	-16,95 (-19,05- -15,10)	0,252

AE: átrio esquerdo; VAEi: volume atrial esquerdo indexado; VE: ventrículo esquerdo; FEVE: fração de ejeção do ventrículo esquerdo; SIV: septo interventricular; DDFVE: diâmetro diastólico final do ventrículo esquerdo; DSFVE: diâmetro sistólico final do ventrículo esquerdo; EPP: espessura da parede posterior; VDFVE: volume diastólico final do ventrículo esquerdo; SLG: Strain Longitudinal Global; LP: lipodistrofia parcial

* p<0,05.

**Figura 2:**
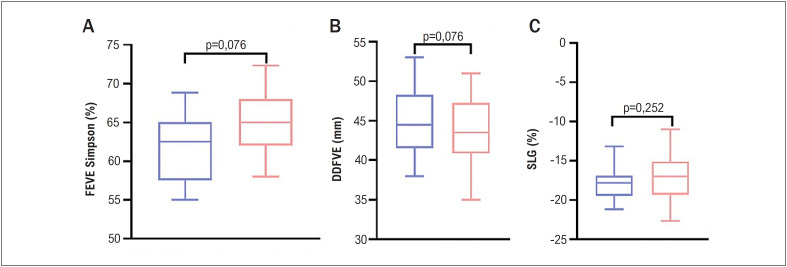
Comparação dos parâmetros ecocardiográficos a função sistólica do ventrículo esquerdo entre os pacientes com lipodistrofia parcial (LP) e Controles (C); FEVE: fração de ejeção do ventrículo esquerdo; DDFVE: diâmetro diastólico final do ventrículo esquerdo; SLG: *strain* longitudinal global.

Em comparação aos controles, os pacientes com LP apresentaram valores mais altos da dimensão do átrio esquerdo (AE), volume atrial esquerdo indexado (VAEi), massa do VE, e espessura da parede posterior (EPP). Ainda, os pacientes com LP apresentaram diferenças nos parâmetros diastólicos do VE, tais como onda E mitral mais baixa, e razão E/A, Doppler tecidual lateral e’ (11,07 ± 3,48 vs. 14,94 ± 2,35 cm/s, p= 0,001), e’ septal (8,0 ± 2,73 vs. 11,38 ± 2,02 cm/s, p= 0,001), e razão E/E’ mais altos. Parâmetros do DDFVE e da função sistólica, como a FEVE, não foram significativamente diferentes entre os grupos. O SLG no *speckle tracking* também não foi significativamente diferente entre os grupos.

Uma proporção significativa dos pacientes com LP apresentou um SLG > -16%, o que significa uma pior função sistólica. Além disso, esses pacientes apresentaram maiores massa do VE, espessura do SIV e EPP, valores mais baixos de DDFVE, e’ lateral, e e’ septal, e uma razão E/e’ elevada (Tabela 3).

**Tabela 3:** Características ecocardiográficas dos pacientes com lipodistrofia parcial, segundo valores do strain longitudinal global (SLG)

Variável	SLG > -16%	SLG < -16%	p
n	12	9	
Dimensão do AE (mm)	36,15 ± 4,547	39 (34,58-41,50)	0,315
VAEi (mL/m^2^)	29,30 (26-30,85)	31 (27,60- 35,89)	0,142
Massa do VE (mL/m^2^)	80,23 (67,29-89,34)	60,69 (51,72-69,27)	0,01*
FEVE Simpson (%)	63,5 (58,50-65)	60,69 (51,72-69,27)	0,316
Espessura do SIV (mm)	11 (10-12)	8 (7-9)	0,003*
DDFVE (mm)	38 (35-39)	44 (40,75-48)	<0,0001*
EPP (mm)	11 (10-12)	9 (8-10)	0,001*
DSFVE (mm)	16,5 (14-22)	20 (15,5- 22,5)	0,99
VDFVE (ml)	52,5 (43-62,5)	56 (46,5-65,5)	0,35
E (cm/s)	60,5 (54-76)	85 (72,5-96,5)	0,02*
A (cm/s)	73 (60-80)	74 (64-82)	0,61
E/a	0,68 (0,49-0,97)	2,24 (1,68-2,87)	0,045*
e' lat (cm/s)	8 (7-9)	12 (10,5-13,5)	<0,0001*
e' sep (cm/s)	6 (5-7)	10 (8,5-12)	<0,0001*
Tempo de desaceleração (mseg)	180 (169-264)	184 (156-267)	0,0067*
e´AVG	7 (6-8,5)	9 (7,25-11,75)	0,002*
E/e´	10,99 (10,33-12,93)	7,46 (6,57-8,83)	<0,0001*

AE: átrio esquerdo; VAEi: volume atrial esquerdo indexado; VE: ventrículo esquerdo; FEVE: fração de ejeção do ventrículo esquerdo; SIV: septo interventricular; DDFVE: diâmetro diastólico final do ventrículo esquerdo; DSFVE: diâmetro sistólico final do ventrículo esquerdo; EPP: espessura da parede posterior; VDFVE: volume diastólico final do ventrículo esquerdo; SLG: Strain Longitudinal Global; LP: lipodistrofia parcial

* p<0,05.

Observou-se uma relação positiva entre as variáveis ecocardiográficas da dimensão do AE (coeficiente *β* 5,6; p<0,001), espessura da parede do VE (coeficiente *β* 1,3, p = 0,011), e’ lateral pelo Doppler tecidual (coeficiente *β* -3,5, p=0,002), e’ septal (coeficiente *β* -3,2, p<0,001) e relação E/e’ (coeficiente *β* 1,5, p=0,021) com o diagnóstico de LP. A relação persistiu estatisticamente significativa após o ajuste para a PAS (Tabela 4).

**Tabela 4:** Associação das variáveis ecocardiográficas e lipodistrofia

	Análise e univariada			Análises multivariadas		
Variável	Coeficiente (*β*)	SE	Valor p	Coeficiente (*β*)	SE	Valor p
Dimensão do AE (mm)	5,6	1,3	<0,001*	5,2	1,6	0,003*
VAEi (mL/m^2^)	3,4	2,7	0,21	2,6	3,1	401
Massa do VE (mL/m^2^)	10,4	4,8	0,08	13,7	6,3	0,038*
FEVE Simpson (%)	1,3	1,5	0,39	1,6	1,7	373
Espessura do SIV (mm)	1,3	0,5	0,011*	1,3	0,6	0,032*
DDFVE (mm)	-1,9	2,1	0,37	-1,8	2,6	0,486
EPP (mm)	1,2	0,4	0,004*	1,2	0,5	0,016*
VDFVE (ml)	5	6,3	0,43	-1,4	7,1	0,849
E (cm/s)	-10,3	5,3	0,06	-8,7	6,1	0,165
A (cm/s)	8,1	5,8	0,17	8	6,5	0,227
e' lat (cm/s)	-3,5	1,1	0,002*	-3,5	1,1	0,003*
e' sep (cm/s)	-3,2	0,8	<0,001*	-3	0,9	0,001*
Tempo de desaceleração (mseg)	13,1	17,1	0,477	4,7	17,3	0,788
e´AVG	-3,5	0,9	<0,001*	-3,3	0,9	0,001*
E/e´	1,5	0,6	0,021*	1,6	0,7	0,035*
SLG (%)	1,1	0,8	0,207	1,6	0,9	0,92

AE: átrio esquerdo; VAEi: volume atrial esquerdo indexado; VE: ventrículo esquerdo; FEVE: fração de ejeção do ventrículo esquerdo; SIV: septo interventricular; DDFVE: diâmetro diastólico final do ventrículo esquerdo; EPP: espessura da parede posterior; VDFVE: volume diastólico final do ventrículo esquerdo; SLG: strain longitudinal global; LP: lipodistrofia parcial

* p<0,05.

## Discussão

O presente estudo mostrou a presença de alterações morfológicas e funcionais em pacientes com LP familiar LP sem sintomas cardiovasculares. Pacientes com LP apresentaram valores mais altos de massa do VE, espessura do VE, e dimensões do AE, bem como índices mais baixos da função diastólica em comparação aos controles. Essas alterações estavam significativamente relacionadas com LP, independentemente dos níveis de PAS dos pacientes. Um número significativo dos pacientes com LP apresentou SLG abaixo dos níveis clinicamente normais (>-16%), apesar da FEVE preservada. Essa remodelação incipiente do VE pode ser similar a outras cardiomiopatias metabólicas, e em alguns casos, progredir para Insuficiência Cardíaca com fração de ejeção preservada (ICFEp).^22^

Apesar de o SLG dos pacientes com LP não ter sido estatisticamente diferente do SLG dos controles, uma proporção significativa de pacientes apresentou um SLG > -16%. Esses pacientes também apresentaram alterações mais pronunciadas da geometria cardíaca, tais como maior massa do VE e sinais de disfunção diastólica. Em nosso conhecimento, nosso estudo é o primeiro a caracterizar o fenótipo cardíaco de um grupo representativo de pacientes com LP.

A LP representa uma manifestação pleomórfica de doenças muito raras que se manifestam como uma redução variável de distribuição de gordura corporal e comprometer o metabolismo.^8^ Os pacientes apresentam resistência à insulina e suas consequências sistêmicas. Os níveis de leptina encontram-se geralmente baixos ou muito baixos.^5^ A LPD generalizada congênita é uma das apresentações mais comuns de LPD e representa um espectro extremo da doença, com uma ausência quase total de tecido adiposo. Um tipo dessa síndrome complexa envolvendo LPD generalizada é a síndrome de Berardinelli-Seip.^23,24^ A LP tipo 2 está usualmente associada com uma mutação no gene LMNA, descrita na população brasileira. A maioria da nossa população foi testada geneticamente e representou um número considerável de casos com mutações documentadas no gene LMNA.

Estudos prévios, muitos com casos isolados, demonstraram diferentes fenótipos de cardiomiopatias em pacientes com LPD, alguns com hipertrofia do VE, mas outros com cardiomiopatia com dilatação do VE.^14,25^ Em um dos estudos,^26^ 44 pacientes com LPD generalizada congênita foram avaliados, e se observou uma alta prevalência de remodelamento hipertrófico do VE e disfunção diastólica, mas nenhum paciente apresentou disfunção sistólica.^26^ Em 2020, Liberato et al.^20^ estudaram uma população brasileira multicêntrica com LPD generalizada congênita. Os autores mostraram que, além de disfunção diastólica (36,6% dos pacientes) e hipertrofia do VE (31,8%), havia uma redução precoce da função sistólica do VE quando avaliada pelo SLG por ecocardiografia com *speckle tracking*. Um SLG reduzido foi positivamente relacionado com os níveis de Ac1Hb glicemia e insulina basal. Em comparação aos nossos dados, poderíamos pensar que a ausência de redução significativa no SLG na LP familiar, quando comparada com LPD generalizada, representa o continuum da disfunção miocárdica no espectro das deficiências do tecido adiposo.

Nosso estudo também reassegurou que esses pacientes apresentam níveis mais altos de pressão arterial, como demonstrado em outras coortes.^13^ Isso poderia ser um fator de confusão, como um gatilho para a hipertrofia miocárdica. Para superar essa limitação, realizamos uma análise univariada e multivariada para analisar os níveis de PAS como um potencial fator de confusão na relação entre LP e parâmetros ecocardiográficos. Assim, considerando-se a PAS, os parâmetros ecocardiográficos de aumento do AE, massa do VE, espessura miocárdica, e marcadores de disfunção diastólica do VE ainda estavam associados com o diagnóstico de LP.

### Implicações clínicas

Os pacientes com LP familiar apresentam maior massa do VE, remodelamento adverso concêntrico, aumento do AE, e disfunção diastólica quando comparados aos controles, mesmo na ausência de sintomas cardíacos. Assim, uma avaliação precoce desses pacientes pode possibilitar um diagnóstico pré-clínico de comprometimento cardíaco. Esse fenótipo cardíaco parece ser similar ao de outras cardiomiopatias, tais como diabetes mellitus, uma causa comum de ICFEp.

### Limitações

Um pequeno tamanho amostral de pacientes com LP é sempre uma limitação de estudos unicêntricos sobre LPD, uma vez que essa é uma doença muito rara. Contudo, nosso número é similar ao de outras publicações, permitindo comparações entre elas.

Os pacientes foram considerados assintomáticos com base nos relatórios de anamnese e na ausência de dispneia nas atividades diárias; no entanto, eles não foram objetivamente testados quanto à capacidade funcional.

Consequências clínicas de cardiomiopatia e informação prognóstica não foram exploradas neste estudo, dado seu delineamento transversal. Os pacientes desta coorte estão sendo seguidos prospectivamente e avaliados quanto aos fatores de risco de eventos cardíacos adversos.

Outra limitação é que nem todos os pacientes foram testados geneticamente.

Por fim, este estudo não foi delineado para explorar a fisiopatologia relacionada à hipertrofia miocárdica nos pacientes com LP.

## Conclusões

Pacientes com LP familiar e sem sintomas cardíacos apresentam alterações tanto da geometria como da função cardíaca. O fenótipo cardíaco corresponde ao de remodelamento ventricular esquerdo, com aumento atrial esquerdo e disfunção diastólica do VE. A FEVE encontra-se ainda preservada, embora alguns pacientes possam apresentar deformação miocárdica sistólica pela análise do SLG. Variáveis ecocardiográficas estão relacionadas ao diagnóstico de LP familiar independente da PAS.
