# Supplementary material for: Alterações Ecocardiográficas da Geometria e da Função Cardíaca em Pacientes com Lipodistrofia Familiar Parcial
Source: Arq Bras Cardiol. 2024 Jun 6;121(6):e20230442. [Article in Portuguese] doi: 10.36660/abc.20230442 (PMC11216334; doi:10.36660/abc.20230442)
Supplement: Translation

# Translation: Echocardiographic Alterations of Cardiac Geometry and Function in Patients with Familial Partial Lipodystrophy

**DOI:** 10.36660/abc.20230442i

**Authors:** Minna Moreira Dias Romano, André Timóteo Sapalo, Natália Rossin Guidorizzi, Henrique Turin Moreira, Paula Ananda Chacon Inês, Lucas Candelária Kalil, Maria Cristina Foss, Francisco José Albuquerque de Paula

**Affiliations:** 1 São Paulo Brazil Centro de Cardiologia da Faculdade de Medicina de Ribeirão Preto – Universidade de São Paulo (USP), São Paulo – Brazil; 2 São Paulo Brazil Divisão de Endocrinologia da Faculdade de Medicina de Ribeirão Preto – Universidade de São Paulo (USP), São Paulo – Brazil

**Keywords:** Lipodystrophy, Heart Function Tests, Echocardiography

## Abstract

**Background:**

Cardiomyopathy associated with partial lipodystrophy (PL) has not been well described yet.

**Objective:**

To characterize cardiac morphology and function in PL.

**Methods:**

Patients with familial PL and controls were prospectively assessed by transthoracic echocardiography and with speckle-tracking echocardiography (global longitudinal strain, GLS). The relationship between echocardiographic variables and PL diagnosis was tested with regression models, considering the effect of systolic blood pressure (SBP). Significance level of 5% was adopted.

**Results:**

Twenty-nine patients with PL were compared to 17 controls. They did not differ in age (p=0.94), gender or body mass index (p= 0.05). Patients with PL had statistically higher SBP (p=0.02) than controls. Also, PL patients had higher left atrial dimension (37.3 ± 4.4 vs. 32.1 ± 4.3 mm, p= 0.001) and left atrial (30.2 ± 7.2 vs. 24.9 ± 9.0 mL/m^2^,p=0.02), left ventricular (LV) mass (79.3 ± 17.4 vs. 67.1 ± 19.4, p=0.02), and reduced diastolic LV parameters (E’ lateral, p= 0.001) (E’ septal, p= 0.001), (E/E’ ratio, p= 0.02). LV ejection fraction (64.7 ± 4.6 vs. 62.2 ± 4.4 %, p= 0.08) and GLS were not statistically different between groups (-17.1 ± 2.7 vs. -18.0 ± 2.0 %, p= 0.25). There was a positive relationship of left atrium (*β* 5.6, p<0.001), posterior wall thickness, (*β* 1.3, p=0.011), E’ lateral (*β* -3.5, p=0.002) and E’ septal (*β* -3.2, p<0.001) with PL diagnosis, even after adjusted for SBP.

**Conclusion:**

LP patients have LV hypertrophy, left atrial enlargement, and LV diastolic dysfunction although preserved LVEF and GLS. Echocardiographic parameters are related to PL diagnosis independent of SBP.

## Introduction

Partial lipodystrophy (PL) is a rare condition characterized by the loss of adipose tissue in a general or partial way.^1^ The prevalence of lipodystrophy is estimated at 1,3 to 4,7 cases per million people and is higher in consanguineous populations.^2^ Its etiology may be congenital^3^ or acquired, and both involve a deficiency of leptin hormone production.^4^ Ectopic deposition of adipose tissue and triglycerides leads to complications such as insulin resistance, diabetes mellitus, hypertriglyceridemia, hepatic steatosis, and increased risk of cardiovascular disease. Familial PL can result from pathogenic variants of the LMNA gene. Although leptin deficiency consequences are still difficult to understand,^5,6^ treatment with exogenous leptin may be highly effective^7,8^ in some cases of generalized lipodystrophy.

Although cardiovascular disease is a significant cause of early death in this population,^9^ cardiomyopathy associated with PL has not been well described yet. Early atherosclerosis, especially in patients with familial PL, may have a prevalence rate of over 60% and manifests before age 45.^10^ The pathophysiological mechanisms seem not only to be dependent on metabolic changes but also on a direct effect of gene mutation on endothelial function.^4,11^ Some cases of lipodystrophy were reported with left ventricular (LV) hypertrophy, others with features of LV dilation, and many were associated with systemic arterial hypertension. Left ventricle remodeling may be associated with pro-inflammatory states,^12-16^ similar to other metabolic conditions related to heart failure with preserved LV ejection fraction.^17^ Patients with PL due to LMNA mutations have an increased risk of arrhythmias.

Echocardiography is a non-invasive tool capable of characterizing cardiac morphologic and functional alterations. Conventional measurements of cardiac chamber dimensions and ventricular systolic and diastolic function, and new techniques to evaluate myocardial deformation, such as speckle tracking echocardiography,^18,19^ were able to detect early myocardial alterations in generalized PL patients,^20^ but were not yet studied in patients with familial PL. Therefore, in this study we aimed to characterize cardiac morphology and initial LV dysfunction in a group of patients with familial PL with no cardiac symptoms.

## Methods

### Study population

This is a cross-sectional study comparing cases and control. A convenience sample of patients with clinical diagnosis of familial PL was invited to perform a prospective echocardiographic analysis. Clinical diagnosis of familial PL was based on a phenotypic presentation of body fat distribution, associated to metabolic abnormalities such as dysglycemia and hypertriglyceridemia.^8^ Some patients, based on clinical indication, had been submitted to genetic testing using Sanger's method or genetic panels (next generation sequencing- NGS panel). Unaffected volunteers, paired (1:1) by sex and age were invited to participate as a control group. Patients were recruited from an endocrinology clinic, where clinical evaluation, and the biochemical and genetic tests were conducted. Patients with PL diagnosis and aged older than 18 years old were invited to participate. Exclusion criteria were patients with poor echocardiography images. Clinical data included age, gender, body mass index (BMI), body surface area (BSA), history of hypertension, systolic blood pressure (SBP), and diastolic arterial pressure (DBP). Laboratory data included total cholesterol, LDL-cholesterol, HDL-cholesterol, triglycerides, fasting glycemia, and HbA1c.

The local ethics committee approved the study (approval number HCRP_3.744.254), and all participants signed a consent form before the study procedures.

### Echocardiography

All transthoracic echocardiographic images were collected prospectively to guarantee the best image quality by the same echo-certified examiner. Images were acquired with Vivid E9 or E95 (GE Healthcare, Horten, Norway), with a phased-array transducer of 1.4-4.6 MHz. Image acquisition strictly followed previously published guidelines.^21^ Briefly, LV apical images were acquired at the longest possible LV axis, thus avoiding the shortening of LV. Images were recorded with electrocardiographic tracings in at least three consecutive cardiac cycles in quiet respiration. All images were acquired at a frame rate of 55-90 frames/sec and analyzed offline with EchoPac software (GE Vingmed Ultrasound AS) version 112. Conventional echocardiographic parameters of LV dimensions and function were collected as follows: left atrium (LA) dimension and volume, LV mass, LV linear dimension at diastole and systole – LV end-diastolic diameter (LVDD), LV end-systolic diameter (LVSD) – LV end-diastolic volume (LVDV), end-systolic volume (LVSV) and ejection fraction (LVEF) derived from modified Simpson's rule; E-and A-waves in Doppler mitral inflow images and deceleration time (DT); and lateral and septal mitral annular diastolic velocities by tissue Doppler echocardiography (E'lateral and E'septal).

### Two-dimensional echocardiography strain analysis

A single experienced physician was responsible for performing two-dimensional strain analyses using the three LV apical views. The second of the three acquired cardiac cycles was chosen for analysis whenever possible. All strain measurements were collected as "full thickness" (or meso) myocardium, and end-systolic values (ESS) were measured, avoiding post-systolic strain measurements. The reference time point was manually defined, at the beginning of the QRS. End-systole was determined at the time of aortic closure defined from Doppler signals at the LV outflow tract when measuring LV global longitudinal strain (GLS). The endocardial border was traced at end-systole, and the region of interest was adjusted to exclude the pericardium. Segments with persistently inadequate tracking were excluded from the analysis. The left ventricle was divided into 18 segments, and a maximum of two excluded segments was deemed tolerable. A cut off value of -16% of GLS was considered to separate subgroups of patients with lipodystrophy (LPD), previously published normal values for this software.^19^ All strain values were expressed as % changes.

### Statistical analysis

Continuous variables with normal distribution were expressed as mean ± standard deviation and those with a non-normal distribution as median and interquartile range (IQR). Distribution of the continuous data were tested for normality using the Shapiro-Wilk test, and equal variances using the Bartlett's test. Categorical variables were expressed as a percentage and frequencies. Comparisons of continuous variables between groups were performed by using unpaired Student's t-test or Mann-Whitney analysis for parametrical and non-parametrical data, respectively, and by the chi-square test for categorical data. Considering that some patients with PL had higher values of SBP than controls in our study, the relationship of echocardiographic variables with PL diagnosis was adjusted for SBP first using the univariate analysis, and then in multivariate linear regression models. All multivariate linear analysis assumptions were met. The significance level was set as p <0.05. All statistical analyses were performed using GraphPad Prism for Windows v9.4.1 or Stata 14.0 (StataCorp, College Station, TX).

## Results

We included 31 patients with PL. However, two patients were excluded because of the poor quality of echocardiographic images. A genetic test was performed in 78.6% of LPD patients, and all of them had variants in the LMNA gene (59.0% R482W, 22.7% R644C, and 18.2%, with an uncertain significance). The control group consisted of 17 unaffected volunteers, paired by age. Baseline characteristics of patients and control group are described in Table 1. PL patients and control group did not differ for age, gender, BMI or BSA. However, as expected, patients with PL showed higher lipid values and glycemia. Patients with PL had statistically higher SBP, but the levels did not meet the diagnostic criteria for hypertension.

**Table 1:** Clinical characteristic of the study population

Variable		Control	PL	p
**n**		17	29	
**Age (y)**		43.84 ± 13.32	44.58 ± 11.77	0.94
**Male gender (%)**		0.00	27.00	
**Weight (Kg)**		64.33 ± 13.69	68.66 ± 17.92	0.41
**BMI (Kg/m^2^)**		24.52 ± 5.04	26.73 ± 4.30	0.05
**BSA (m^2^)**		1.676 ± 0.16	1.70 ± 0.23	0.9
**Pathogenic Variant**				
	r482w	0	12	
	r644c	0	5	
**Clinical charcacteristic**				
	Glycemia (mg/dl)	88.39 (83.50-96.00)	128.50 (89.06-206)	<0.0001*
	HBA1C (%)	5.45 (4.92-5.87)	8.70 (5.8-11.40)	<0.0001*
	HDL (mg/dl)	55.00 (40.50-61.25)	35.61 (31.04-42.32)	0.01*
	LDL (mg/dl)	104 (84-142)	110.00 (70.50-163.65)	0.58
	TG(mg/dl)	86 (67.50-124)	221.80 (162.00-406.40)	<0.0001*
	SBP (mmHg)	111.00 (101-117)	123.5 (112-134)	0.02*
	DBP (mmHg)	69 (62-72)	75(66-82)	0.1
**Medication (%)**				
	Insulin	0	51.7	
	Metformin	0	41.37	
	Gliclazide	0	3.44	
	Statin	0	41.37	
	Fibrates	0	68.96	
	Anti-hipertensive	5	68.96	

BMI: body mass index; BSA: body surface area; SBP: systolic blood presure; DBP: diastolic blood pressure; PL: partial lipodistrophy.

Echocardiographic conventional parameters of cardiac geometry and LV function are presented in Table 2, and some variables are expressed as a graphic in Figures 1 and 2.

**Table 2:** Echocardiographic characteristics of population

Variable	Control	PL	p
LA dimension (mm)	32 (28.5-33.75)	38 (34.25-40.00)	0.001*
LAVi (ml/m^2^)	26.26 (20.41-30.89)	30 (26-32.90)	0.023*
LVmass i(ml/m^2^)	67.54 (49.60-79.79)	77.34 (67.21-88.29)	0.022*
LVEF Simpson (%)	63.50 (59.25-65.75)	64.50 (61-67.50)	0.77
IVS thickness (mm)	8.00 (7-9)	9 (8-11)	0.003*
LVDD (mm)	44.50 (40.50-48.50)	43 (41-47)	0.526
PWT (mm)	7.5 (6.50-8.87)	9 (8-10)	0.001*
LVSD (mm)	21.25 (18.13- 24)	20 (15-25)	0.985
LVDV (ml)	57 (47.93-63)	56 (45.50-77.50)	0.345
E (cm/s)	82 (69-96)	74 (54-83)	0.016*
A (cm/s)	59 (48-73)	69 (53-80)	0.197
E/a	0.891 (0.61-1.54)	2.19 (1.68-3.54)	0.035*
e' lat (cm/s)	15 (12-17)	11 (9-14)	<0.0001
e' sep (cm/s)	10 (10-14)	8 (6-11)	<0.0001
Deceleration time (mseg)	186 (175.3-217.3)	180 (162-244)	0.45
e´AVG	12.5 (11.5-15.5)	9 (7-12)	<0.0001
E/e´	6.19 (5.15-7.55)	7.38 (6-9.1)	0.017*
GLS (%)	-18.05 (-19.78- - 17.09)	-16.95 (-19.05- -15.10)	0.252

LA: left atrial; LAVi: LA volume indexed; Lvmass: LV mass; LV: left ventricle; LVEF: left ventricle ejection fraction; IVS: interventricular septum; PWT: posterior wall thickness; LVSD: left ventricular systolic dimension; LVDD: left ventricular end-diastolic diameter; LVSD: left ventricular end-sistolic diameter; LVDV: left ventricular end-diastolic volume; PL: partial lipodystrophy.

* =p<0.05.

**Figure 1:** Comparison of echocardiographic parameters of cardiac geometry and left ventricular (LV) diastolic function between partial lipodystrophy (PL) patients and controls; LA: left atrial; LAVi: Left atrium volume index.

**Figure 2:** Comparison of echocardiographic parameters of left ventricular (LV) systolic function between partial lipodystrophy (PL) patients and controls; LVEF: left ventricular ejection fraction; LVDD: left ventricular diastolic dimension; GLS: global longitudinal strain.

PL patients differed from control with higher left atrial (LA) dimension, LA indexed volume (LAi), LV mass, interventricular septum (IVS) thickness, and posterior wall thickness (PWT). Also, compared to the control group, PL patients showed differences in LV diastolic parameters such as lower mitral E wave, and higher E/A ratio, lower tissue Doppler e’ lateral (11.07 ± 3.48 vs. 14.94 ± 2.35 cm/s, p= 0.001), e'septal (8.0 ± 2.73 vs. 11.38 ± 2.02 cm/s, p= 0.001), and E/E’ ratio. Parameters of LVDD, and systolic function as LVEF were not significantly different between groups. Speckle tracking GLS also showed no significant difference between groups.

A significant proportion of PL patients presented with GLS > -16% which means a worse systolic function. Also, these patients had a higher LV mass, IVS thickness and PWT, lower LVDD, and lower values of e’ lateral, e’ septal, and elevated E/e’ ratio (Table 3).

**Table 3:** Echocardiographic characteristics of partial lipodystrophy patients, according to global longitudinal strain (GLS) values

Variable	GLS > -16%	GLS < -16%	p
n	12	9	
LA dimension (mm)	36.15 ± 4.547	39 (34.58-41.50)	0.315
LAVi (mL/m2)	29.30 (26-30.85)	31 (27.60- 35.89)	0.142
LV mass i(mg/m2)	80.23 (67.29-89.34)	60.69 (51.72-69.27)	0.01*
LVEF Simpson (%)	63.5 (58.50-65)	60.69 (51.72-69.27)	0.316
IVS thickness (mm)	11 (10-12)	8 (7-9)	0.003*
LVDD (mm)	38 (35-39)	44 (40.75-48)	<0.0001*
PWT (mm)	11 (10-12)	9 (8-10)	0.001*
LVSD (mm)	16.5 (14-22)	20 (15.5- 22.5)	0.99
LVDV (ml)	52.5 (43-62.5)	56 (46.5-65.5)	0.35
E (cm/s)	60.5 (54-76)	85 (72.5-96.5)	0.02*
A (cm/s)	73 (60-80)	74 (64-82)	0.61
E/a	0.68 (0.49-0.97)	2.24 (1.68-2.87)	0.045*
e' lat (cm/s)	8 (7-9)	12 (10.5-13.5)	<0.0001*
e' sep (cm/s)	6 (5-7)	10 (8.5-12)	<0.0001*
Deceleration time (mseg)	180 (169-264)	184 (156-267)	0.0067*
e´AVG	7 (6-8.5)	9 (7.25-11.75)	0.002*
E/e´	10.99 (10.33-12.93)	7.46 (6.57-8.83)	<0.0001*

LA: left atrial; LAVi: LA volume index; LV: left ventricular; LVEF: left ventricular ejection fraction; IVS: interventricular septum; PWT: posterior wall thickness; LVSD: left ventricular systolic diameter; LVDD: left ventricular end-diastolic diameter; LVDV: left ventricular end-diastolic volume; LPD: lipodystrophy.

* =p<0.05.

There was a positive relationship of echocardiographic variables of LA dimension (*β* coefficient 5.6, p<0.001), LV wall thickness (coefficient 1.3, p = 0.011), tissue doppler e’ lateral (*β* coefficient -3.5, p=0.002), e’ septal (*β* coefficient -3.2, p<0.001) and E/e’ relation (*β* coefficient 1.5, p=0.021) to PL diagnosis. The relation persisted as statistically significant after adjusting for SBP (Table 4).

**Table 4:** Association of echocardiographic variables and Lipodystrophy

	Univariate Analyses			Multivariate analyses		
Variable	Coefficient (*β*)	SE	p-value	Coefficient (*β*)	SE	p-value
LA dimension (mm)	5.6	1.3	<0.001*	5.2	1.6	0.003*
LAVi (mL/m^2^)	3.4	2.7	0.21	2.6	3.1	401
LV mass (mg/m^2^)	10.4	4.8	0.08	13.7	6.3	0.038*
LVEF Simpson (%)	1.3	1.5	0.39	1.6	1.7	373
IVS thickness (mm)	1.3	0.5	0.011*	1.3	0.6	0.032*
LVDD (mm)	-1.9	2.1	0.37	-1.8	2.6	0.486
PWT (mm)	1.2	0.4	0.004*	1.2	0.5	0.016*
LVDV (mL)	5	6.3	0.43	-1.4	7.1	0.849
E (cm/s)	-10.3	5.3	0.06	-8.7	6.1	0.165
A (cm/s)	8.1	5.8	0.17	8	6.5	0.227
e' lat (cm/s)	-3.5	1.1	0.002*	-3.5	1.1	0.003*
e' sep (cm/s)	-3.2	0.8	<0.001*	-3	0.9	0.001*
Deceleration time (mseg)	13.1	17.1	0.477	4.7	17.3	0.788
e´AVG	-3.5	0.9	<0.001*	-3.3	0.9	0.001*
E/e´	1.5	0.6	0.021*	1.6	0.7	0.035*
GLS (%)	1.1	0.8	0.207	1.6	0.9	0.92

LA: left atrial; LAVi: LA volume index; LV: left ventricular; LVEF: left ventricle ejection fraction; IVS: interventricular septum; PWT: thickness of posterior wall; LVSD: left ventricle systolic dimension; LVDD: left ventricular end-diastolic diameter; LVDV: left ventricular end-diastolic volume; LPD: lipodystrophy.

* =p<0.05.

## Discussion

This study showed that cardiac morphological and functional alterations are present in patients with familial PL with no cardiovascular symptoms. Patients with PL presented higher LV mass, LV thickness, and LA dimensions, as well as lower indices of diastolic function when compared to control. These abnormalities were significantly related to PL, independently of patients’ SBP levels. A significant number of PL patients presented with GLS below clinical normal levels (> -16%), despite preserved LVEF. This incipient LV remodelation may be similar to other metabolic cardiomyopathies, and in some cases progress to heart failure with preserved ejection fraction (HFpEF).^22^

Although GLS of PL patients was not statistically different from control, a significant proportion of patients presented GLS > -16%; these patients also had more pronounced alterations of cardiac geometry, such as higher LV mass and signs of diastolic dysfunction. To our knowledge, our study is the first to characterize the cardiac phenotype in a representative group of familial PL patients.

PL represents a pleomorphic manifestation of very rare diseases which manifest as a variable reduction of body fat distribution and compromise in metabolism.^8^ Patients present insulin resistance and its systemic consequences. Leptin levels are usually low or very low.^5^ Congenital generalized LPD is one of the most common presentations of LPD and represents an extreme spectrum of disease, with an almost total absence of adipose tissue. One type of this complex syndrome involving generalized lipodystrophy is Berardinelli-Seip syndrome, described in the Brazilian population.^23,24^ Type 2 PL is usually associated with LMNA gene mutation. Most of our population was genetically tested and represented a considerable number of cases with documented LMNA gene mutations.

Previous studies, many of them with isolated cases, have demonstrated different phenotypes of cardiomyopathies in patients with LPD, some with LV hypertrophy, but others with dilated LV cardiomyopathy.^14,25^ One of them^26^ studied 44 patients with congenital generalized LPD and showed a high prevalence of hypertrophic LV remodeling and diastolic dysfunction, but none presented systolic dysfunction.^26^ In 2020, Liberato et al.^20^ studied a multicentric Brazilian population with congenital generalized LPD. They showed that, besides diastolic dysfunction (36.6% of patients) and LV hypertrophy (31.8%), there was an early reduction of LV systolic function when evaluated by GLS speckle tracking echocardiography. Reduced GLS was positively related to Ac1Hb levels, glycemia, and basal insulin. Compared to our data, we could think that the lack of significant reduction in GLS in familial PL, when compared to generalized LPD, represents the continuum of myocardial dysfunction in the spectrum of adipose tissue deficiencies.

Our study also reassured that these patients have higher arterial pressure levels, as demonstrated in other cohorts.^13^ This could be a confounder as a trigger to myocardium hypertrophy. To overcome this limitation, we performed univariate and multivariate analysis to account for SBP levels as a potential confounder in the relationship between PL and echocardiographic parameters. So, even when considering SBP, echocardiographic parameters of LA enlargement, LV mass, myocardial thickness, and LV diastolic dysfunction markers were still associated with PL diagnosis.

### Clinical implications

Patients with familial PL present higher LV mass, concentric adverse remodeling, LA enlargement, and diastolic dysfunction compared to control, even in the absence of cardiac symptoms. Thus, an early examination of PL patients may allow a pre-clinical diagnosis of cardiac impairment. This cardiac phenotype seems to be similar to other metabolic cardiomyopathies, such as diabetes mellitus, a common cause of HFpEF.

### Limitations

A small sample size of patients with PL is always a limitation of single-center studies on LPD, as the disease is very rare. However, our number is similar to other publications, allowing comparisons between them.

Patients were considered asymptomatic based on medical history reports, and absence of dyspnea to daily activities; however, patients were not objectively tested for functional capacity.

Clinical consequences of cardiomyopathy and prognostic information were not explored in our study, given its cross-sectional design. Patients of this cohort are prospectively followed and assessed for risk factors of adverse cardiac events.

Another limitation is that not all patients were genetically tested.

Finally, this study was not designed to explore the pathophysiology related to myocardial hypertrophy in PL patients.

## Conclusions

Patients with familial PL and no cardiac symptoms present cardiac geometry and function alterations. Cardiac phenotype follows LV remodeling, with left atrial enlargement and LV diastolic dysfunction. LVEF is still preserved, although some patients may show reduced LV myocardial systolic deformation by GLS analysis. Echocardiographic variables are related to familial PL diagnosis independent of SBP.
